# Advances in Extracellular Matrix Metalloproteinases: Implications for Renal Cell Carcinoma Pathophysiology, Diagnostics, and Therapeutics

**DOI:** 10.3390/medicines13010009

**Published:** 2026-03-03

**Authors:** Evangelia Krikou, Ioanna A. Anastasiou, Panagiotis Sarantis, Hariklia Gakiopoulou, Irini Theochari, Dimitra Grigoriadou, Andreas C. Lazaris, Eleftheria Lakiotaki

**Affiliations:** 1 First Department of Pathology, School of Medicine, National and Kapodistrian University of Athens, 75 Mikras Asias Str., Building 10, Goudi, 11527 Athens, Greece; evangeliakrikou@gmail.com (E.K.); charagak28@gmail.com (H.G.); theoirene@hotmail.com (I.T.); dgrigoriadou@med.uoa.gr (D.G.); alazaris@med.uoa.gr (A.C.L.); ellakiotaki@gmail.com (E.L.); 2 Diabetes Center, First Department of Propaedeutic Internal Medicine, Medical School, National and Kapodistrian University of Athens, Laiko General Hospital, Goudi, 11527 Athens, Greece; 3 Department of Pharmacology, Medical School, National and Kapodistrian University of Athens, 11527 Athens, Greece; 4 Molecular Oncology Unit, Department of Biological Chemistry, Medical School, National and Kapodistrian University of Athens, 11527 Athens, Greece

**Keywords:** renal cell carcinoma, extracellular matrix, matrix metalloproteinases, clear cell RCC, tumor microenvironment

## Abstract

Renal cell carcinoma (RCC) is a common tumor that heavily depends on extracellular matrix (ECM) remodeling, an essential process involved not only in normal tissue homeostasis but also in malignant growth. This article reviews the role of matrix metalloproteinases (MMPs, zinc-dependent endopeptidases) in matrix degradation and ECM reorganization in the setting of RCC. We focus on the specific role of MMP2, MMP7, and MMP9 in clear cell renal cell carcinoma (ccRCC) and major subtypes of RCC. Higher levels of these MMPs are associated with high-grade tumors, increased risk of metastasis, and poorer patient survival rates, indicating that they may have value as prognostic markers. This review also discusses how ECM composition and structure are altered in the tumor microenvironment (TME), thereby preventing cell interactions and promoting cancer growth. Finally, it compiles the existing studies to anticipate a future era in which MMPs could serve as effective prognostic biomarkers and potential treatment targets for RCC, with implications for improving diagnostic and therapeutic interventions targeting ECM remodeling to suppress cancer progression.

## 1. Introduction

Renal cell carcinoma (RCC) is one of the most prevalent cancers, with 434,840 estimated new cases in 2022 worldwide [1]. It ranks as the sixth and ninth most common cancer in men and women in the United States, respectively. Clear cell RCC (ccRCC) is the most common type, comprising 75–80%, and is molecularly defined by inactivation of the von Hippel–Lindau (VHL) tumor suppressor gene [2,3].

Seventy percent of tumors are diagnosed incidentally, and most (80%) are stage I. RCC mortality decreased by 2% annually from 2016 to 2020, despite an increasing incidence (1%/year) between 2015 and 2019. Patients diagnosed with renal masses should consult a urologist. Ablation, surgery, or simply watchful waiting are all treatment options. For tumors less than 4.0 cm, partial nephrectomy achieves a five-year survival rate of more than 94%. Immunotherapy can be effective for advanced RCC, with response rates of 42–71% and a median survival rate of 46–56 months [1,4].

Matrix metalloproteinases (MMPs) are a family of endopeptidases, which are Zn-dependent and play an essential role in the degradation and modification of the extracellular matrix (ECM) [5]. This activity is critical for tissue remodeling, but it also facilitates tumor cell mobility, invasion, and the activation of the pro-tumoral microenvironment [6].

In ccRCC, studies have focused on MMP2, MMP7, and MMP9 because of their critical roles in tumor development [5,6]. Elevated MMP7 levels have been associated with poor survival outcomes, suggesting its potential as a prognostic biomarker. Likewise, higher levels of MMP9 have also been associated with more aggressive and metastatic tumors, so it is not clear whether the protein is related to unfavorable prognosis. These results suggest that MMPs not only promote ccRCC development but may also be promising targets for prognostic and therapeutic purposes [7,8].

This review aims to delineate the role of MMPs in ECM degradation and restructuring in RCC.

## 2. Literature Search

This review entailed a comprehensive literature search employing the PubMed database, focusing on the keywords “Renal cell carcinoma” and “Matrix Metalloproteinases”. The search included 289 articles that were published between January 1962 and December 2025. The inclusion criteria comprised peer-reviewed articles published in English, including human observational studies (cohort, case–control, registry, and biobank) and interventional studies, as well as substantial meta-analyses and systematic reviews. Animal or in vitro studies were included if they offered substantial mechanistic insights into renal cell carcinoma. Exclusion criteria encompassed conference abstracts, non-peer-reviewed literature, and 12 articles deemed insufficiently informative, thereby ensuring that only studies directly relevant to the topic were evaluated. We also checked the reference lists of the included manuscripts to find additional related studies. The study’s relevance was determined by its congruence with the review’s objectives, whereas methodological quality was assessed by emphasizing contemporary and representative research. The narrative format of this review may introduce selection bias; however, it enabled the integration of diverse mechanistic, translational, and epidemiological evidence that is frequently neglected by traditional systematic review methodologies.

## 3. Extracellular Matrix (ECM)

### 3.1. Normal Tissue Reshaping

The primary purpose of the ECM is to maintain tissue health, helping cells grow and differentiate, and tissues remain intact [9]. Each tissue or organ has its own ECM. Loose connective tissue contains reticular fibers and ground substances, for instance, while bone ECM is heavy with collagen fibers and mineral deposits. Blood plasma, which serves as the internal environment for circulating blood cells, is also an ECM. The complex mixture of ECM components, often rich in carbohydrates to manage hydration, and also containing protein fibers to control elasticity and structure, plays essential roles in regulating water, cell communication, and tissue health. ECM controls conditions that could harm cell structure and provides an environment in which cells can live [9].

The expansion of the tumor is driven by unrestrained growth, leading to heterogeneity in cell types and creating a hostile microenvironment impenetrable to anti-tumor agents and immune cells [10]. The combined effects of bulk acellular and cellular components in the TME, cells with accelerated cell cycles and/or suppressed cell death, orchestrated by the ECM, result in uncontrolled cancer growth [10].

ECM composition can have diverse effects on cell proliferation; for instance, radiation-induced ECM regulation can alter cell cycle arrest and progression in both malignant cells [11,12] and fibroblasts [13]. While acellular components secreted by depositional cells promote oncogenic progression, they may also drive tumor cells into a quiescent state, thereby acquiring stem-like properties and increasing resistance to environmental stress during cell division and metastasis. For example, type III collagen helps in keeping dormant cancers in development, creating a niche for secondary growth [14].

Therefore, ECM behaves as a dynamic signaling center and a ‘between space’ for local biochemistry, integrating mechanotransduction signals from both cancerous and normal cells, as well as other bioactive molecules that control tumor tissue proliferation [15].

### 3.2. MMPs

MMPs, which were initially identified in the 1960s for their collagenolytic activity during tadpole tail resorption [16,17], are now recognized as a family of endopeptidases under the metzincin superfamily called matrixins. All these enzymes are highly homologous, multidomain, zinc (Zn^2+^)-containing metalloproteinases [18] capable of degrading diverse components of the connective tissue protein matrix.

Each typical MMP structure covers a propeptide (80 amino acids), a catalytic metalloproteinase domain (170 amino acids), a linkage site between the two of variable length (called “hinge region”), and a hemopexin region of about 200 amino acids in length.

Hemopexins belong to the AML protein family, which includes membrane-type matrix metalloproteinases (MT-MMPs) [18,19,20]. The cysteine switch motif (PRCGPVD) contains a central chain region that forms a disulfide bridge with a Zn^2+^ ion bound to it. This chelated sulfhydryl group maintains MMPs in the inactive zymogen form [21,22,23].

MMPs contain a zinc-binding motif (HEXXHXXGXXH) that not only is present in the catalytic domain but also serves as a site for Zn^2+^ coordination; three histidines, a glutamate, and a specific Met turn sequence (XBMX) are found to chelate each of the Zn^2+^ ions [21]. In vertebrates, the MMP family comprises 28 members, of which at least 23 are expressed in humans, and 14 are found only in the human vasculature [22].

MMPs are classified according to substrate specificity and structural domain organization into collagenases, gelatinases, stromelysins, matrilysins, MT-MMPs, and others [24]. These classes have specific structural features that distinguish them from the prototypical MMP structure [23]. The detailed backbone conformation of MMP13 has been described previously [17].

In RCC, quick breaking down of the ECM is linked to the tumor being more invasive and the patient’s prognosis being worse [25]. The main driver of this process is the upregulation of enzymes such as MMPs, which degrade key components of the ECM and facilitate tumor cell motility and invasion of adjacent tissues [6]. ECM molecule content in molecules such as collagen IV, laminin, and fibronectin often differs in RCC tissue compared with normal kidney tissue [26,27]. For example, if collagen IV and laminin are degraded or altered, the basement membrane can be damaged. This phenomenon promotes cancer cell invasiveness. Changes in fibronectin expression favor tumor cell adhesiveness, motility, and signaling capacity, all of which promote tumor growth [26,27]. These changes in the ECM that support tumor invasion and spread are linked to aggressive disease and worse clinical outcomes. As a result, the state of ECM components and their regulatory enzymes is now considered an important factor in tumor malignant potential and a potential treatment target in RCC.

## 4. Prevalence, Epidemiology, Clinical Features, Staging, and Prognosis of RCC

In 2020, around 431,288 new cases of kidney cancer (KC) were diagnosed globally [28]. The primary subtypes of RCC are ccRCC (approximately 70% of cases), papillary carcinoma (pRCC; 10–15%), and chromophobe carcinoma (RCC; 5%) [29]. Subtypes with an occurrence of <1% are rare and typically not included in most studies [30].

While there is some indication that these histologic subgroups may differ in clinical behavior, response to therapy, and genetic characteristics, the lack of detailed epidemiological information has limited our ability to study their comparative patterns in a more sophisticated way [30]. ccRCC is the most frequent and most intensively investigated subtype of RCC, accounting for approximately 75–80% of all cases [31].

The fundamental molecular lesion in ccRCC is the inactivation of the VHL gene on chromosome 3p25. The loss or mutation of this gene results in increased stabilization of hypoxia-inducible factors (HIFs), chief among them HIF-1α and HIF-2α, under normoxic conditions. Stabilization consequently leads to the overexpression of many genes involved in angiogenesis, such as vascular endothelial growth factor (VEGF), platelet-derived growth factor (PDGF), and other factors involved in blood vessel formation and metastasis.

Additional mutations in other genes, such as PBRM1, SETD2, BAP1, and TP53, contribute to the genetic heterogeneity and aggressive potential of ccRCC, with chromosomal losses, particularly 3p, being a particularly characteristic example [32,33].

Clinically, RCC presents with no symptoms in the early stages and is incidentally discovered during abdominal imaging for other reasons [34]. If symptoms do appear, they usually include flank pain, hematuria, a palpable mass, weight loss, or constitutional symptoms such as fatigue or fever. Tumor secretion of various chemokines may elicit paraneoplastic syndromes, including hypercalcemia.

The diagnosis of RCC relies heavily on imaging studies, such as contrast-enhanced computed tomography (CT) scans. Such scans typically show a well-defined, circumscribed renal mass. Magnetic resonance imaging (MRI) can provide further information in selected cases. In some cases, especially when the clinical presentation is atypical or in inoperable cases, a biopsy may help confirm the diagnosis [34].

Staging is important for determining prognosis and guiding therapy; accordingly, the American Joint Committee on Cancer (AJCC) 8th edition TNM staging system has become the standard. Tumors at an early stage (Stage I), confined to the kidney and no more than 7 cm in size, have a 5-year survival rate of over 90% [35,36]. Stage II tumors are larger but confined to the kidney and show survival rates around 70–75%.

Stage III tumors involve infiltration of the renal vein or perinephric tissues or lymph node metastasis, with survival rates dropping to around 50–60%. Stage IV tumors show distant metastases or extensive local infiltration, and five-year survival rates are less than 20%.

**Figure 1:**
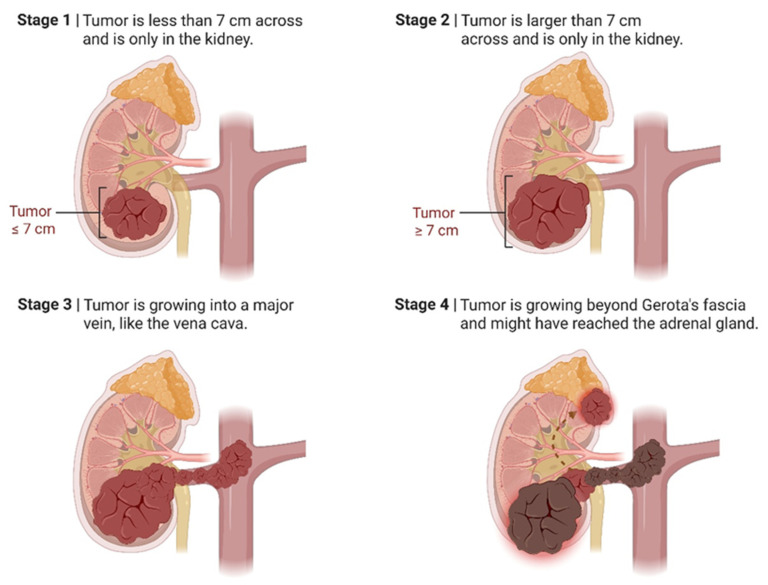
Stages of Renal Cell Carcinoma Progression. The image illustrates the four stages of kidney cancer. At Stage 1, the tumor is ≤7 cm in size and confined to the kidney. During Stage 2, the tumor is bigger than 7 cm but still confined to the kidney. Stage 3 is when the tumor has invaded into adjacent prominent veins, such as the vena cava, and may be invading the surrounding tissue, but remains within Gerota’s fascia. In Stage 4, the cancer has expanded outside of Gerota’s fascia into tissues, such as the adrenal gland, and possibly to distant lymph nodes or other areas beyond the solar plexus. Created in BioRender. ANASTASIOU, I. (2025) https://BioRender.com/c00mfbd. accessed on 17 October 2025.

The outcome often depends not only on stage but also on other factors, such as tumor histologic subtype, tumor size, nuclear grade (Fuhrman/ISUP), presence of necrosis, molecular markers, and genetic features [35,36]. As shown in Figure 1, the course of RCC at different stages depends on tumor size and tumor spread.

## 5. Current Treatment Strategies in Clear Cell Renal Cell Carcinoma

Therapeutic strategies differ between localized and metastatic disease [37]. For localized disease, partial nephrectomy is the state of the art and preferred for tumors less than 7 cm. Large or central tumors call for radical nephrectomy. In some instances, less-invasive interventions, such as cryo- or radiofrequency ablation, can be considered, especially in small, isolated tumors and with patients who suffer from substantial comorbidities that would increase the surgical risk. Active surveillance may be appropriate for small, low-grade tumors in elderly patients or patients in poor general health [37].

Systemic chemotherapy in locally advanced and metastatic ccRCC focuses on maintaining the disease under control and extending survival [38]. In the past, cytokine therapies such as interferon-alpha and interleukin-2 were of little benefit and showed substantial toxicity [39]. For quite some time, tyrosine kinase inhibitors (TKIs) have been a cornerstone of the treatment of metastatic RCC, with, e.g., sunitinib being the preferred first-line treatment [38]. The introduction of targeted therapies changed the natural history of the disease, and agents that target the VEGF pathway, sunitinib, pazopanib, cabozantinib, and axitinib, have been shown to block the tumor’s ability to create new blood vessels. mTOR inhibitors like everolimus and temsirolimus have also been crucial for their anti-proliferative and cell growth pathway-targeting effects. Although effective, these therapies are mainly palliative [38,39].

The most recent breakthrough for the management of ccRCC is the combination of immunotherapy with targeted therapy [40]. Checkpoint inhibitors, such as nivolumab (a PD-1 blocker), have been shown to extend overall survival in metastatic settings and are approved by the FDA, and combining them with ipilimumab (a CTLA-4 inhibitor) offers durable responses for intermediate- and poor-risk patients [41]. The combination of angiogenesis inhibitors with immunotherapy, for instance, axitinib or cabozantinib, has shown improved efficacy compared with monotherapy and has become standard treatment today [42]. Current research is investigating new agents, combination regimens, and biomarkers to predict response and further personalize therapy [40,43].

Overall, ccRCC is best managed through multimodal care that combines surgery, systemic treatment, and radiotherapy to meet the needs of individual patients based on their stage of disease, genetic profile, and overall health [44]. The biologically unpredictable behavior of ccRCC, which can be indolent in some cases and aggressive in others, remains a therapeutic problem. Current research priorities focus significantly on the molecular underpinnings of tumor heterogeneity, resistance, and the immune microenvironment to enhance the therapeutic efficacy of personalized treatments. Advances in molecular diagnostics and genomic profiling, which are closely linked to precision medicine, will help identify patients who would benefit most from newly developed agents or immunotherapies. In addition, novel immunomodulatory agents and cytokine therapies, as well as vaccines and adoptive cell therapies, are being studied in new clinical trials to improve long-term outcomes and achieve more durable remissions or cures [44].

Furthermore, the role of nephron-sparing surgery in selected patients underscores the importance of preserving renal function, which is increasingly recognized as associated with prolonged survival and better quality of life [45]. These patients should undergo regular image-based surveillance to detect recurrence/metastases. Platinum-based therapy is often employed for these patients, and supportive care, including paraneoplastic syndrome treatment and management of side effects related to systemic therapies, is crucial to maintaining the patient’s quality of life throughout the course of their disease [45].

## 6. MMPs and TIMPs in RCC Progression

At the cell level, there is considerable heterogeneity in tumor excretory products among different histologic types of RCC [46,47,48]. Nevertheless, all RCCs studied display defects in lacunae integrity and assembly. They can be considered mechanisms by which tumors may evade immune surveillance and regulate the normal cellular microenvironment [46,47,48].

MMPs can degrade almost any ECM component, including collagen, elastin, and fibronectin. As a rule, these enzymes are secreted into the extracellular matrix in an inactive form, requiring activation to become functionally active [49]. High levels of MMPs have been implicated in invasion, angiogenesis, tumor development, and resistance to existing anticancer therapies in some solid tumors [50].

To prevent excessive tissue destruction and maintain ECM organization, mature metalloproteinases are tightly regulated by a family of four endogenous proteins known as tissue inhibitors of metalloproteinases (TIMPs), which bind to active MMPs and inactivate them [51,52,53]. TIMPs are also involved in processes such as tumor growth, apoptosis, and angiogenesis [54]. TIMPs are all secreted, but only TIMP3 is found in the extracellular matrix [55]. Glycosylation of TIMP3 would, in theory, be an impediment to glycan-associated MMP binding, but instead detracts from its potency, and cancer is a disease replete with abnormal glycosylation [56]. While TIMP3 may act by preventing MMP activity, suppressing tumor growth, angiogenesis, invasion, and metastasis, TIMP3 also has other cellular functions unrelated to MMP inhibition, such as inducing apoptosis in the TNF response and inhibiting the migration and proliferation of endothelial cells [57]. However, in contrast to the upregulation of TIMP-1, there is abundant evidence that loss of TIMP3 expression is common across a wide variety of human cancers [58,59]. Proteomic analysis suggests that certain MMPs serve as biomarkers for disease progression [60]. A study showed TIMP1 was significantly upregulated in RCC tissues and cell lines. High TIMP1 expression was significantly associated with poor prognosis and served as an independent prognostic factor [61]. In vitro, TIMP1 knockdown reduced RCC cell proliferation, migration, and invasion, while its overexpression enhanced these processes.

Furthermore, TIMP1 promoted RCC progression through the epithelial–mesenchymal transition (EMT) pathway. Thus, TIMP1 could be a critical biomarker for diagnostic purposes in RCC and may be introduced as a prognostic indicator [61]. Research found that in ccRCC, MMP7 expression was significantly higher, whereas TIMP2 expression was lower compared to controls [62]. Both markers correlated with tumor grade and stage, and both MMP7 upregulation and TIMP2 downregulation were associated with disease progression. MMP7 was an independent prognostic factor, indicating that it and TIMP2 might function as biomarkers for ccRCC prognosis [62].

**Figure 2:**
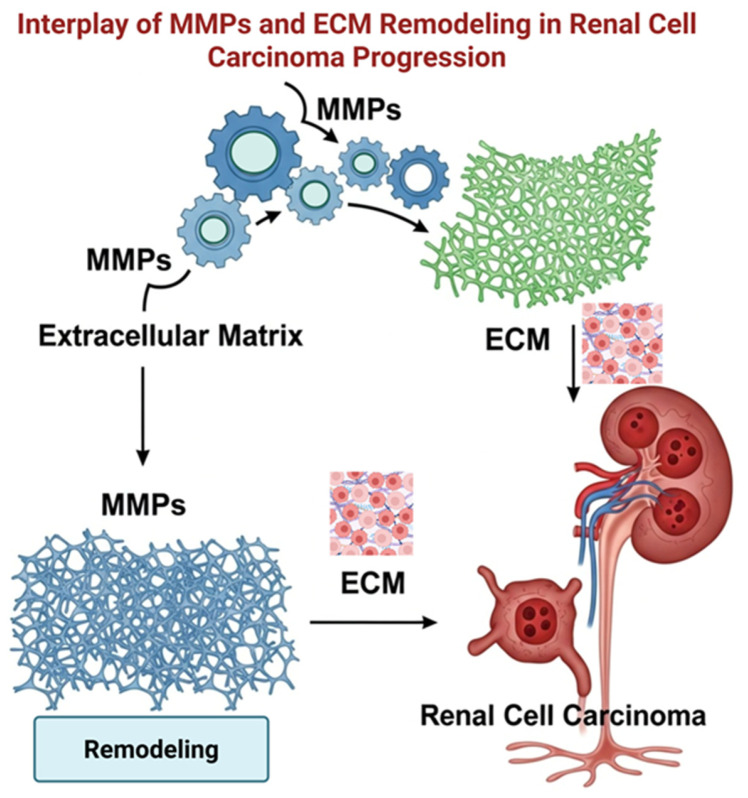
A detailed schematic representation of MMPs and the remodeling of the ECM, illustrating their crucial roles in the progression of RCC. This diagram highlights the mechanisms by which MMPs facilitate ECM degradation, thereby contributing to tumor growth, invasion, and metastasis and ultimately influencing the severity and progression of RCC. Created in BioRender. Anastasiou IA. https://BioRender.com/7qowaze, accessed on 9 February 2026. ECM: Extracellular matrix; MMPs: Matrix metalloproteinases; RCC: Renal cell carcinoma.

Despite these findings, the clinical significance of measuring MMP mRNA or protein levels remains to be fully determined, mainly because data are lacking on which forms of the enzyme are biochemically active. For instance, in serum samples from individuals receiving targeted therapy with agents such as sunitinib, circulating levels of MMPs, such as MMP9 and TIMP2, have been measured [63]. Figure 2 presents a comprehensive scheme illustrating the roles of MMPs and ECM remodeling in RCC progression. This diagram emphasizes how MMPs facilitate the degradation of ECM components, thereby promoting tumor growth, invasion, and metastasis, ultimately influencing the aggressiveness of RCC and impacting patient prognosis. Although individual concentrations of MMP9 and TIMP2 often do not change significantly with disease progression, the MMP9/TIMP2 ratio is high, suggesting it may be a predictive biomarker of therapy response. Table 1 summarizes treatment strategies and biomarker expressions in RCC.

### MMP2, MMP7, and MMP9

In the last two decades, research has focused on the prognostic significance of MMP expression and activity in RCC. MMP2 and MMP9 are known as gelatinases because they degrade basement membrane components, such as type IV collagen, elastin, and fibronectin, which are important substrates for both tumor invasion and angiogenesis [49]. An imbalance favoring MMP activity over TIMP inhibition leads to excessive ECM degradation, promoting tumor progression. RT-PCR analyses have shown that the MMP2/TIMP2 ratio is often higher in advanced RCC than in adjacent normal tissue [64].

In another experiment, researchers test the predictive value of immunohistochemical distribution patterns of TIMP2, MMP2, and MMP9 for stage and survival in colorectal tumors [65]. However, analyses of 212 frozen tumor sections showed that TIMP2 expression with differential localization (basement membrane vs. diffuse stromal staining) was more often associated with localized than with regional or distant metastases. These trends were associated with improved survival, although none were statistically significant. Expression of MMP2 and MMP9 was not related to tumor stage or survival. High content of MMP9-positive macrophages was associated with poor differentiation, whereas weak staining of MMP2 in epithelium approached significance for an association with differentiation. In summary, TIMP2 levels differentiate between localized and metastasizing colorectal cancer but do not have the power to predict micrometastases in curatively resected patients. MMP2 and MMP9 expression provided limited information in tumor staging and prediction [65].

One study used semi-quantitative RT-PCR to analyze the expression levels of MMP2, MMP9, MT1-MMP, TIMP1, and TIMP2 in 76 cases of RCC of various histologic types [66]. The results showed that Expression of all five genes was significantly higher in RCC tissues than in normal renal tissue (*p* < 0.05), and gene expression correlated to some extent with tumor size (T) and lymph node status (N). There were no significant correlations with histologic type or tumor grade. However, at advanced tumor stage, expression levels of MMP2, MMP9, and MT1-MMP are all significantly higher, suggesting a relationship between increased gene activity and tumor progression. Consequently, these findings suggest that higher Expression or activity for these matrix metalloproteases and their inhibitors is linked to RCC growth and progression [66].

In one study, researchers assessed MMP7 and MMP2 expression in patients with RCC and examined their relationships with clinical and pathological data [67]. Immunohistochemistry was performed on tumor samples from 20 patients with RCC who underwent surgery at Osaka City University Medical School Hospital. The results revealed that MMP2 Expression was significantly higher in advanced-stage and high-grade tumors than in early-stage and low-grade tumors. However, in contrast to MMP2, there was no significant difference in MMP7 expression levels between early and advanced stage tumors (*p* = 0.1859). The increased levels of MMP7 in high-grade RCC suggest a potential role in tumor growth and metastasis, highlighting the distinct roles MMP2 and MMP7 can play in the progression of RCC. From this perspective, such observations might aid in the development of targeted therapies against RCC [67].

Elevated MMP7 levels in high-grade and invasive RCC may be related to its role in tumor cell detachment, basement membrane invasion, and infiltration into surrounding tissues [62]. This linkage supports its function as a regulatory molecule inherent to living organisms. Rapid quantitative molecular detection technologies are beginning to play a role in the treatment of various diseases. It is only the lack of ultrasensitive testing for MMP7 upregulation that retains MMP7 as a current research topic. Higher levels of MMP7 upregulation were associated with worse treatment outcomes and worse overall survival in this study [62].

The MMP2 protein expression levels in these samples were assessed by immunohistochemistry, and the findings were verified using a meta-analysis with CMA 2.0 software [68]. MMP2 positivity was significantly higher in RCC tissues than in normal tissues (*p* < 0.001). Higher MMP2 expression was associated with large tumors (>5 cm), lymph node migration (LNM), high tumor grade (poorly differentiated), and advanced stages (III-IV), with statistical differences in each case (*p* < 0.05). Furthermore, individuals with positive MMP2 expression had a significantly higher 5-year survival rate than those with negative MMP2 expression (*p* = 0.037). These data are supported by meta-analysis, underscoring their reliability. In short, MMP2 expression is related to tumor size, grade, and stage, as well as lymphatic spread in RCC, suggesting its potential as a crucial prognostic biomarker for disease progression and patient prognosis [68].

Using cDNA arrays, the expression of the MMP superfamily was studied in RCC [69]. MMP2 and MMP9 activity in 178 patients with RCC, classified by the Heidelberg system, was quantified. MMP2 and MMP9 transcript levels were assessed in 145 samples, including 16 fresh-frozen specimens. In 16 fresh-frozen samples from newly diagnosed RCC cases, gelatinolytic activity was assessed by zymography. Additionally, MMP2, MMP9, MMP11, MMP14, and MMP16 were all upregulated in conventional (clear cell) RCC compared to chromophobe RCC. MMP1, MMP11, and MMP16 expressions were more pronounced in papillary RCC than in conventional RCC. There was a clear correlation between MMP9 transcript levels and enzymatic activity (*p* = 0.001), which was related to worse disease-free survival (*p* = 0.001) and an increased risk of migration (*p* = 0.011). Gelatinolytic activity aligned with MMP9 mRNA expression, showing higher activity in ccRCCs irrespective of stage or tumor grade (*p* = 0.001). MMP9 was identified as a significant prognostic predictor in multiple survival analysis models (*p* = 0.0054). Notably, compared with its mRNA levels, MMP2 enzyme activity in RCC tissues was absent, suggesting post-transcriptional regulation [69].

For RCC prognosis, MMP9 shows that differential transcriptional regulation, coupled with its association with aggressive features such as migration or worse outcomes, offers promise as an effective biomarker. In contrast, MMP2 appears to be regulated by some mechanism other than transcription, the details of which remain unknown [69]. Additionally, MMP9 contributes to angiogenesis by releasing angiogenic factors from the subcutaneous interstitial matrix.

MMP9 is the most significant MMP for RCC, as it shows robust correlations with aggressive disease characteristics and prognosis. Its enzymatic activity and functions in the process of cancer render it potent not only as a prognostic marker but also as a target for therapy. Although MMP2 is strongly associated with advanced disease stage, its functional constraints may prevent it from serving as an independent prognostic marker. For MMP7, the evidence is inferior, and therefore it should not be emphasized to the same extent as for MMP2 and MMP9.

A comparative and integrative review of the roles of MMPs and TIMPs in RCC progression identifies areas of agreement and conflicting results. Levels of MMP2, MMP7, and especially MMP9 are always higher in RCC tissues than in normal kidney tissues. MMP9 has the strongest and most consistent connection to aggressive disease traits and a dire outlook for patients. MMP2 is frequently associated with advanced tumor stage and metastatic potential; however, its predictive capacity appears to be comparatively weaker. Some studies have found that its mRNA expression and enzymatic activity are different. This could be because of post-transcriptional regulation. MMP7, although higher in aggressive tumors, does not reliably distinguish between early and advanced RCC, suggesting inconsistencies in detection methods or limited sample sizes. Differences in study design, such as using mRNA assays instead of protein assays, measuring active versus total enzyme levels, or differences in patient groups, such as tumor subtype and disease stage, often lead to different research results. The interaction between MMPs and their inhibitors, TIMPs, particularly the increase in TIMP1 levels and decrease in TIMP2 levels, complicates their roles as biomarkers and therapeutic targets. These inconsistencies underscore the imperative for standardized methodologies and well-defined patient cohorts to clarify the prognostic and predictive relevance of MMPs and TIMPs in RCC, thereby enriching the discourse and guiding future research. Table 2 compares MMP2, MMP7, and MMP9 features in terms of their prognostic significance based on expression levels and activity in RCC.

## 7. Challenges in Renal Cell Carcinoma Research and Treatment

Despite significant advancements in the study and treatment of RCC in the past decade, numerous challenges persist. For example, although targeted agents such as TKIs have demonstrated the ability to extend survival in ccRCC, it is necessary to maximize their effectiveness and develop strategies to circumvent resistance. Prospective investigation of patient-specific factors and tumor biology is necessary to improve the efficacy of these therapies, leading to more individualized treatment strategies and better overall outcomes for patients [70].

A two-way crosstalk between ccRCC cells and the perinephric adipose tissue (PAT) was reported, wherein ccRCC secreted parathyroid hormone-related protein (PTHrP) to stimulate browning of the PAT through protein kinase A (PKA) activation [71]. The PAT-elicited thermogenesis led to excessive lactate release and facilitated ccRCC proliferation, invasion, and migration. The cyclin-dependent kinase inhibitors were capable of worsening this cycle by further promoting PAT browning, as observed in the context of ccRCC treatment. The anti-tumor effect of the TKI sunitinib was then further enhanced when the adipocyte browning was pharmacologically inhibited using H89 or KT5720. These findings underscore the importance of ccRCC-PAT crosstalk and support combination therapies to improve TKI efficacy [71].

One study prepared lysolipid-containing thermosensitive liposomes (LTSLs) for targeted delivery of MATT to the TME [72]. The LTSLs exhibited a rapid release profile of the encapsulated contents at 42 °C compared with vehicle controls, and increased tumor distribution with MATT-LTSLs led to approximately 20-fold suppression of in vivo growth in 4T1 tumor-bearing mice. In vivo, there was also a 50% and 43% reduction in MMP2 and MMP9 activity, respectively, associated with a 30% and 43% decrease in their Expression by MATT-LTSLs. In addition, metastatic lung nodules and tumor microvessels decreased by 7- and 6-fold, respectively, following MATT-LTSL therapy. These findings indicate a synergistic approach to metastasis inhibition and demonstrate the potential of a combination of a cytotoxic agent and MATT in the treatment of metastatic cancer [72].

Emerging resistance gradually exhausts treatment efficacy, and therefore, additional treatment measures are required. Immunotherapeutics also led to the establishment of immunotherapy as the standard treatment for RCC, but they only partially modulate immune responses [29]. These poor response rates highlight the pressing need for more effective methods to elicit an immune response against RCC.

The existing chemotherapeutic drugs kill all dividing cells in the body indiscriminately. They may cause damage to normal tissues and common side effects, including hair loss, nausea, and blood disorders [73,74]. These have also been the side effects of less selective MMP inhibitors. The past few years have seen a variety of strategies for localized drug-delivery systems and tumor-specific targeting [73,74]. Despite combining various monoclonal antibodies with conventional chemotherapy, the outcomes in quality of life and survival have been modestly improved. In an effort to evade systemic chemotherapy, additional studies are exploring antibody-drug conjugate matrices in which the exclusive binding of monoclonal antibodies is used to deliver highly potent cytotoxic reagents [75]. The linkers in such systems are usually flexible tethering molecules that bridge antibodies and drugs, enabling conjugates to retain circulation stability while triggering drug release upon internalization into target cells. For instance, a liposomal delivery system has been described recently, in which an antinucleosome monoclonal Ab is linked to a cell-penetrating Tat peptide via an MMP2-cleavable sequence [76]. This design biases the antibody to recognize the tumor specifically and to induce drug release via MMP-2 activity, followed by cell uptake [76]. Such a design enabled the antibody to specifically recognize tumor cells and induce drug release via MMP2 activity, thereby facilitating uptake [76]. Mesoporous silica-based nanoparticles have also been developed, and the chemotherapeutic drug doxorubicin is actively targeted to tumor cells via MMPs and other receptors [77]. Other developments include chitosan nanoparticles that silence MMP3 and MMP13 in chondrocytes to prevent dedifferentiation during autologous cartilage transplantation [78]. Furthermore, an injectable MMP-degradable hydrogel for the delivery of recombinant tissue inhibitor of MMP3 post-myocardial infarction was recently found to prevent detrimental cardiac remodeling in a swine model, as a localized, not systemic, treatment with no significant impact on healthy tissues [79].

The necessity of diagnostic procedures that are both more effective and less invasive is immense. Such advances might also allow early detection of more aggressive diseases, real-time tracking of treatment responses, and improved clinical decision-making. By delivering early, precise information, these advanced diagnostics would enable healthcare professionals to tailor better treatment strategies that benefit patients and improve efficiency along the care pathway.

## 8. Conclusions

This review aimed to provide a comprehensive overview of the current understanding of matrix metalloproteinases (MMPs) in renal cell carcinoma (RCC); however, certain limitations must be acknowledged. The studies chosen for this narrative review were selected based on their perceived relevance, not through a systematic search, which could have introduced selection bias. Moreover, many of the included studies are observational and retrospective, making them more susceptible to confounding and recall bias.

One important problem with using MMPs as biomarkers is their inherent activity fluctuations. We still do not know whether measuring MMP mRNA or protein levels is clinically significant because we do not know much about the procedure’s feasibility or how other factors, such as impaired kidney function, affect systemic concentrations. For example, elevated MMP levels in the blood could indicate an aggressive tumor. However, they could also reflect low kidney infiltration, tissue damage from surgery or treatment, or extensive tumor spread, making them hard to interpret and assess [80].

Preventing MMPs from functioning is a promising way to keep RCC tumors from spreading and metastasizing. Current research focuses on developing selective MMP inhibitors that specifically target tumor-associated activity while safeguarding healthy tissues. Combining MMP inhibition with well-established treatments such as TKIs and immune checkpoint inhibitors might make treatment more effective by reducing invasiveness and possibly regulating drug resistance [81]. Researchers are working on finding circulating MMPs as a less invasive way to diagnose RCC. However, right now, the evidence shows that urinary MMP activity is not sensitive or specific enough to diagnose RCC because its levels vary among healthy people and there are no clear differences between patients and controls. So, MMPs have not yet been proven to be non-invasive biomarkers for RCC, and their use in the clinic is still being studied.

Future research should focus on strict, forward-looking studies to confirm the clinical usefulness of MMPs as diagnostic and therapeutic targets. More specifically, efforts should be made to make the regulation of MMPs clearer, such as by finding specific isoforms and studying epigenetic mechanisms. This will help make treatment plans that are unique to each patient and improve the results for RCC patients. Until this kind of proof is found, MMP-based tests or treatments should only be used with care in clinical settings.

## Figures and Tables

**Table 1:** Summary of treatment strategies and biomarker expressions in RCC.

Treatment	Expression in RCC	Association with Tumor Characteristics	Role in Disease Management	Benefits/Prognostic Value	References
Cryo- or Radiofrequency Ablation	N/A	Considered for small, isolated tumors in high-risk patients	Less invasive treatment option	Preserves renal function	[37]
TKIs	High Expression (Sunitinib)	Standard treatment for metastatic ccRCC	Controls disease, extends survival	Often used as a first-line treatment	[38]
mTOR Inhibitors	High Expression (Everolimus, Temsirolimus)	Important for targeting cell growth pathways	Anti-proliferative effects	Mainly palliative, but it can aid in disease management.	[38,39]
Checkpoint Inhibitors	High Expression (e.g., Nivolumab, Ipilimumab)	Effective in metastatic settings	Enhances immune response against tumors	Improves overall survival in combination therapy	[40]
Angiogenesis Inhibitors	High Expression (Axitinib, Cabozantinib)	Combined with immunotherapy for better efficacy	Blocks tumor blood vessel formation	Standard treatment, improved outcomes vs. monotherapy	[43]
MMPs	Elevated in ccRCC	Correlates with tumor grade and stage	Invasion and resistance to therapies	Independent prognostic factor	[62]
TIMPs (TIMP1, TIMP2)	TIMP1 upregulated, TIMP2 downregulated	TIMP1 is associated with poor prognosis; TIMP2 correlates with progression	It inhibits MMPs and impacts tumor growth and apoptosis.	TIMP1 is a critical biomarker; TIMP2 is linked to prognosis	[61]

v: Clear cell RCC; TIMPs: Tissue inhibitors of metalloproteinases; TKIs: Tyrosine kinase inhibitors; N/A: not available.

**Table 2:** Comparison of MMP2, MMP7, and MMP9 in RCC.

Matrix Metalloproteinase	Expression in RCC	Association with Tumor Characteristics	Role in Disease Progression	Prognostic Value	References
MMP2	Higher Expression in RCC compared to normal tissue (*p* < 0.001)	Associated with larger tumors, lymph node metastasis, high grade, and advanced stages (III-IV) (*p* < 0.05)	Linked to tumor growth, invasion, and lymphatic spread	Positive Expression correlated with a better 5-year survival rate (*p* = 0.037)	[49,64,65,68]
MMP7	Increased levels in high-grade and invasive RCC; no significant early vs. advanced stage difference (*p* = 0.1859)	Elevated in high-grade tumors, indicating a potential role in tumor growth and metastasis	May facilitate tumor cell detachment and invasion	Higher levels are associated with worse treatment outcomes and overall survival	[62,67]
MMP9	Upregulated in RCC compared to normal tissue; significant correlation between mRNA levels and enzymatic activity (*p* = 0.001)	Related to worse disease-free survival and increased risk of metastasis (*p* = 0.011)	Contributes to angiogenesis and tumor aggressiveness	Identified as a significant prognostic predictor (*p* = 0.0054)	[65,66,69]

MMP: Matrix metalloproteinase.

## Data Availability

No new data were created or analyzed in this study. Data sharing is not applicable to this article.

## Abbreviations

The following abbreviations are used in this manuscript: AJCC: American Joint Committee on Cancer; ccRCC: clear cell RCC; CT: tomography; ECM: extracellular matrix; HIFs: hypoxia-inducible factors; KC: kidney cancer; LNM: lymph node metastasis; LTSLs: lysolipid-containing thermosensitive liposomes; MMPs: matrix metalloproteinases; MRI: magnetic resonance imaging; MT-MMPs: membrane-type matrix metalloproteinases; PAT: perinephric adipose tissue; PDGF: platelet-derived growth factor; PKA: protein kinase A; pRCC; papillary carcinoma; PTHrP: parathyroid hormone-related protein; RCC: renal cell carcinoma; RCT: randomized controlled trial; TIMPs: tissue inhibitors of metalloproteinases; TKIs: tyrosine kinase inhibitors; TME: tumor microenvironment; VEGF: vascular endothelial growth factor; VHL: von Hippel–Lindau.
